# Sickle cell β-thalassemia diagnosed at age 40: a case report

**DOI:** 10.1007/s00277-025-06483-y

**Published:** 2025-07-01

**Authors:** Christos G. Nikolaidis, Despoina Gyriki, Dimitrios G. Gogos, Elisavet Stavropoulou

**Affiliations:** 1 Department of Internal Medicine, Vostaneio-General Hospital of Mytilene, Mytilene, Greece; 2 Department of Hematology, Vostaneio-General Hospital of Mytilene, Mytilene, Greece; 3 Master Program in “Food, Nutrition and Microbiome”, Laboratory of Hygiene and Environmental Protection, Department of Medicine, Democritus University of Thrace, Alexandroupolis, Greece; 4 Infectious Diseases Service, Department of Medicine, Lausanne University Hospital, University of Lausanne, Lausanne, Switzerland

**Keywords:** Sickle cell β-thalassemia, HbSβ-thal, Hemoglobinopathy, Late-onset, Age, Case report

## Abstract

Hereditary hemoglobinopathies, including sickle cell disease and thalassemias, affect thousands of newborns annually, predominantly in low-and middle-income countries. Sickle cell β-thalassemia (HbSβ-thal), a form of compound heterozygosity involving β-thalassemia, presents with a wide range of clinical severity depending on the specific mutations. However, the clinical manifestations remain poorly defined. We report the case of a 40-year-old Greek female patient presenting with symptomatic sickle cell β-thalassemia, symptoms of tissue hypoperfusion caused by markedly low hemoglobin levels and notably, bone marrow necrosis. Remarkably, her condition remained undiagnosed until her admission to the emergency department. This case underscores the importance of maintaining a high index of clinical suspicion for the late-onset diagnosis of HbSβ-thal, particularly considering its increased prevalence in certain countries. The successful treatment strategy employed in this case highlights the critical role of individualized care in managing the severe and multifaceted symptoms associated with this genetic disorder, offering valuable insights for clinicians worldwide.

## Introduction

Each year, thousands of babies are born with hemoglobinopathies, with more than 500,000 babies born with sickle cell disease (SCD) and almost 60,000 affected by clinically significant thalassemias, exhibiting varying clinical presentations based on inherited alleles [1]. Among them, 30,000 are transfusion dependent [2]. Over 80% of these instances take place in the tropical belt of low- and middle-income nations [3].

SCD is a common inherited condition caused by a mutation in beta-globin (Glu6Val) [4]. It includes a number of genetic variants, such as homozygous HbSS and compound heterozygous forms like HbSC and HbSβ-thalassemia. When combined with the beta-thalassemia mutation, the severity depends on the extent of hemoglobin A (HbA) production impairment (with HbSβ⁺-thal varying from severe to mild) [5]. Table 1 describes typical blood counts and hemoglobin electrophoresis patterns commonly seen in individuals with different types of SCD [6].

**Table 1:** Laboratory characteristics and clinical presentation of common sickle cell genotypes (hematological parameters represent typical median values) [6]

Genotype	Hb (g/dL)	MCV (fL)	Reticulocyte (%)	HbS (%)	HbA (%)	HbF (%)	Clinical Phenotype
HbAS (sickle trait)	12.5–16	80–95	1–2	≤ 40	> 60	< 1.0	Normal
HbSS (sickle cell anemia)	8.3 (6.0–10.0)	90 (85–100)	11.8 (8–15)	> 90	0 (0)	< 20 (5–20)	Severe
HbSβ0 thalassemia	8.9 (7.5–10)	72 (60–80)	10 (8–15)	> 80	0 (0)	< 20	Severe
HbSβ + thalassemia	10.8 (8.5–13)	68 (60–75)	4.1 (3–8)	> 60	10–30	< 20	Mild-Moderate
HbSC	10.6 (9–12)	77 (75–95)	4.2 (2–7)	50 (45–60)	0 (0)	0 (0–3)	Moderate

Cardiovascular and hepatic-related complications are the leading causes of mortality in patients with SCD and thalassemias, followed by infections, especially in patients who have undergone splenectomy or who have impaired splenic function due to multiple vaso-occlusive crises [7, 8]. Patients with HbSβ0 thalassemia are reported to have a high mortality rate, with deaths resulting from acute splenic sequestration, septicemia, cerebral abscess, renal failure, acute chest syndrome in pregnancy, bone marrow embolism, and sudden deaths alongside complications like gallstones, aplastic crises, leg ulcers, and septicemia. HbSβ + thalassemia shows a wide spectrum of manifestations, ranging from severe cases to milder forms with fewer complications [9].

This report describes a complex case of compound heterozygosity for SCD with β-thalassemia, first diagnosed at the age of 40, manifesting with severe anemia, symptoms of myocardial hypoperfusion, and bone marrow necrosis.

## Case presentation

A 40-year-old Caucasian woman presented to the emergency department with severe back pain, unresponsive to painkillers. She had a medical history of glucose-6-phosphate dehydrogenase (G6PD) deficiency, hypothyroidism, and smoking. Also, she occasionally used a combination of codeine, paracetamol and caffeine for headaches.

Blood tests revealed hypochromic microcytic anemia with an Hgb level of 10.2 g/dL (normal range for females: 12–16 g/dL), a mean corpuscular volume (MCV) of 67.2 fL (normal range: 79–98 fL), and mildly elevated levels of lactate dehydrogenase (LDH), C-reactive protein (CRP), and liver enzymes, along with a high D-dimer level. Computed tomography angiography (CTA) of the abdominal aorta was performed to rule out an acute vascular event, which was clinically suspected due to the patient’s severe back pain. The imaging revealed no pathological findings. The patient was administered tramadol following guidance by a neurosurgeon, showed improvement, and was discharged from the emergency department.

After seven days, the patient returned to the emergency department presenting with symptoms of dizziness, nausea, difficulty concentrating, and palpitations. Clinical examination revealed pallor, tachypnea, and tachycardia. The digital rectal examination was negative for gastrointestinal bleeding. The electrocardiogram showed sinus rhythm tachycardia and findings suggestive of myocardial ischemia, specifically inverted T waves and downsloping ST-segment depression, mainly in leads V3-V6. ABGs revealed respiratory alkalosis (pH: 7.487, pCO_2_: 16.1 mmHg, pO_2_: 88.2 mmHg, HCO_3_: 12.3 mmol/L) and high lactates: 4.8 mmol/L.

Initial blood tests revealed an Hgb value of 3.1 g/dL, a significant rise in CRP, ferritin, reticulocytes, and LDH levels, and impaired liver function tests. Further detailed information on laboratory findings, including the evolution of key parameters across multiple visits, from emergency department admission to follow-up, is presented in Table 2.

**Table 2:** Evolution of laboratory findings across multiple visits, including emergency department admission and Follow-up

Blood Tests	Normal Values	2013	2015	Initial Visit (02/2024)	Second Visit (pre-transfusion) (03/2024)	Discharge	Follow-up (6 months)
White Blood Cells (x10³/µL)	4.5–10.5	4.7	6.5	8	6.8	**3.4**	**3.9**
Neutrophils (/µL)	1.8–7	1.8	4.7	6.9	3.6	1.7	2.6
Lymphocytes (/µL)	1.2–3.8	2.1	1.3	0.7	2.8	1.4	**1.0**
NRBC (/µL)	0	-	-	-	**2.3**	-	**-**
Red Blood Cells (x10^6^/µL)	3.9–5.5	4.13	5.20	4.64	**1.38**	**3.36**	**3.64**
Hemoglobin (g/dL)	12–16	13	**11.6**	**10.2**	**3.1**	**8.9**	**10.2**
Hematocrit (%)	36–46	38.9	36.7	**31.1**	**9.5**	**28.1**	**31.5**
MCV (fL)	79–98	94.2	**70.6**	**67.2**	**68.5**	83.8	86.3
MCH (pg)	26–32	31.6	**22.2**	**21.9**	**22.4**	26.5	27.9
Platelets (x10³/µL)	140–440	276	401	**502**	**123**	**149**	313
Reticulocytes (%)	0.5–2.5	-	-	-	**3.97**	**5.49**	-
Indirect Bilirubin (mg/dL)	0.1–1	-	-	-	1	**-**	-
Direct Bilirubin (mg/dL)	0–0.5	-	-	-	**0.72**	**-**	-
Total Bilirubin (mg/dL)	0.3–1.2	-	-	-	**1.70**	0.92	0.81
Troponin-lhs (pg/mL)	< 15.6	-	-	-	7	**-**	-
Aspartate Aminotransferase (U/L)	5–33	12	18	**37**	**65**	15	21
Alanine Aminotransferase (U/L)	5–31	15	17	**46**	**85**	28	17
γ-Glutamyl Transferase (U/L)	< 36	-	-	19	**43**	**177**	10
Alkaline Phosphatase (U/L)	< 150	-	-	55	71	118	135
LDH (IU/L)	< 248	-	-	-	**3066**	**633**	237
rea (mg/dL)	< 55	-	-	21	**93**	19	20
Creatinine (mg/dL)	0.6–1.09	-	-	0.88	1.01	0.85	0.78
C-Reactive Protein (mg/L)	0–7	**21.21**	-	**10.90**	**160.30**	**82.50**	< 1
Prothrombin Time- INR	0.8–1.2	-	-	1.02	0.99	0.97	-
aPTT (sec)	25.1–37.7	-	-	33.3	**23.9**	27.3	-
Fibrinogen (mg/dL)	170–420	-	-	**616**	**447**	**532**	-
Ferritin (ng/mL)	13–150	-	135	-	**> 4744**	-	**159**
d-Dimers (mg/L)	< 500	-	-	5.44	**6.22**	**10.86**	**-**

*NRBC *Nucleated Red Blood Cells, *MCV *Mean corpuscular volume, *LDH *Lactate dehydrogenase, *aPTT *activated partial thromboplastin time

The examination of the peripheral blood smear revealed anisocytosis, poikilocytosis, microcytosis, hypochromia, blister cells, numerous fragmented red blood cells, and a few red blood cells resembling sickle cells. Additionally, there were numerous erythroblasts, 200 per 100 nucleated cells. Urine tests indicated the presence of leukocytes and hemoglobin. Abdominal ultrasound and chest X-rays revealed no acute findings, prompting further evaluation by a hematologist.

### Diagnosis, treatment, and outcome

The patient was admitted to the internal medicine department for further investigation. Because of anemia with concomitant pain, an extensive differential diagnosis for hematologic, cardiovascular, pulmonary, and infectious causes was conducted. Acute hemorrhage was unlikely due to the absence of overt bleeding signs and negative digital rectal examination. Severe iron deficiency anemia was considered given the microcytic morphology and symptoms such as pallor and fatigue but the elevated ferritin levels and high reticulocyte count were inconsistent with this diagnosis. Hemoglobinopathies, such as thalassemias, were also included in the differential diagnosis because of the patient’s origin from a high- prevalence Greek island, microcytic and hypochromic erythrocytes, possible sickle cells seen in the peripheral blood smear, as well as bone pain, which can be attributed to marrow expansion or bone infarcts. A blood sample was collected for hemoglobin electrophoresis. Acute hemolytic anemia was also part of the differential diagnosis due to high LDH levels, reticulocytosis, and significant presence of erythroblasts, as well as numerous fragmented red blood cells on peripheral blood smear. Regarding immune-mediated hemolysis, there was no evidence of spherocytosis on the peripheral blood smear. Direct antiglobulin testing (DAT-Coombs) was therefore performed. Moreover, although the patient had a medical history of G6PD deficiency, no bite cells or Heinz bodies were observed on the peripheral smear, and there was no identifiable oxidative trigger at the time of presentation. Other red cell disorders such as pyruvate kinase deficiency, hereditary spherocytosis, hereditary elliptocytosis and paroxysmal nocturnal hemoglobinuria (PNH) were considered but were less likely given the lack of supporting clinical or laboratory findings. Microangiopathic hemolytic anemias (MAHA)—including Thrombotic Thrombocytopenic Purpura (TTP), Hemolytic Uremic Syndrome (HUS), and Diffuse Intravacular Coagulation (DIC)—were excluded based on normal platelet counts, absence of schistocytes, and lack of organ dysfunction. Other causes of hemolysis, such as medication and toxics were excluded by medical history. Of note, the patient was not pregnant. Hemophagocytic syndrome/Hemophagocytic lymphohistiocytosis (HLH) was also considered in the differential diagnosis given the markedly elevated ferritin, LDH, liver enzyme abnormalities as well as elevated C - reactive protein and d-dimers. However, no other cytopenias were present, and C-reactive protein was not very high. Similarly, bone marrow disorders were suspected, but the peripheral blood smear was not suggestive, no other cytopenias were present. A bone marrow aspiration and biopsy were ultimately performed. Concerning suspicion of myocardial ischemia, ECG findings were secondary to profound anemia and troponin levels were low. Due to the marked anemia and associated ECG abnormalities secondary to hypoperfusion, the patient was urgently transfused with a total of four units of red blood cells (RBC) and supported with intravenous fluids (normal saline). Moreover, since suspicion of autoimmune hemolytic anemia (AIHA) was raised, she was empirically started on emergency methylprednisone treatment, in accordance with evidence that glucocorticoids not only abrogate immune‑mediated hemolysis but also have direct actions to enhance erythroid progenitor proliferation and erythropoietin production, thereby increasing erythropoiesis [10]. Also, she was started on piperacillin-tazobactam for a suspected concomitant urinary tract infection, given the back pain, which could suggest a positive Giordano sign, and urine culture showing growth of *Escherichia coli*.

Blood samples for complete blood count and hemoglobin electrophoresis were obtained at admission (prior to any transfusion) when the patient’s hemoglobin was 3.1 g/dl. Pre-transfusion testing later revealed negative direct antiglobulin test (DAT), excluding warm AIHA.

The detailed hemoglobin electrophoresis results, performed prior to blood transfusion, revealed the following:

- Hemoglobin F (HbF): 1.3%.
- Hemoglobin A (HbA): 48.8%.
- Hemoglobin A2 (HbA2): 3.4%.
- Hemoglobin S (HbS): 40.2%.

These findings indicated a significant alteration in the hemoglobin profile, consistent with a complex hemoglobinopathy.

Further genotype and molecular analysis was performed using a peripheral blood sample. The results showed no pathogenic variants in the α-globin gene. However, the β-globin gene analysis identified the pathogenic variant NM_000518.5(HBB): c.20 A > T (p.Glu7Val), responsible for the production of hemoglobin S, as well as the pathogenic variant IVS-I-6 (T > C) [NM_000518.5(HBB): c.92 + 6 T > C], in compound heterozygosity. These findings confirmed the diagnosis of SCD with compound heterozygosity for sickle cell/β-thalassemia.

As part of the anemia’s investigation workflow, a bone marrow aspiration and biopsy were performed, revealing marrow necrosis, likely of ischemic type. Bone marrow necrosis was explained by ischemia secondary to severe anemia and vascular occlusion. Moreover, abdominal ultrasound and computed tomography (CT) imaging were performed, which showed no splenic atrophy or other pathological findings.

Hydroxyurea was the cornerstone of the patient’s treatment following the confirmation of sickle cell/β-thalassemia. The treatment plan also included hematopoietic vitamins (folic acid and vitamin E), magnesium supplements, analgesics (paracetamol and opioid preparations), and low molecular weight heparin. Notably, as aforementioned, due to an initial suspicion of autoimmune hemolytic anemia, methylprednisolone was administered before the results of direct antiglobulin test (DAT/direct Coombs) were available. Following the negative results, the corticosteroid treatment was gradually tapered and subsequently discontinued.

The patient was discharged after 15 days of hospitalization and remains healthy 1 year post discharge.

## Discussion

Greece has one of the highest prevalences of hereditary hemoglobinopathies, with 7.5% of the population being thalassemia carriers. HbSβ-thalassemia is the most common sickling syndrome, and βS/β0 is the most frequent genotype, presenting similarly to SCD. The national registry of hemoglobinopathies in Greece reported 4032 patients with hemoglobinopathies, with 2099 patients with thalassemia major and 1032 patients with SCD [7].

National screening policies introduced in the 1970 s have facilitated early diagnosis [7, 11]. Despite these advances, SCD and thalassemias are not currently included in routine neonatal screening in Greece, which currently covers only phenylketonuria, G6PD deficiency, galactosemia, and congenital hypothyroidism [12]. Extended screening may be conducted by private laboratories, typically at the physician’s discretion or patient request, but it is not reimbursed. Comprehensive screening is generally performed in the context of genetic counseling for both parents and fetuses [7, 13].

Greek molecular defects are very heterogeneous; the most common is the IVS1-nt 110 defect, followed by the CD39 and IVS1-nt 6 genotypes14 [14]. The mutation responsible for the β-thalassemia discussed in this case occurs frequently in Portuguese and Mediterranean populations and is commonly known as “Portuguese” β-thalassemia. Previous studies have shown that the β + IVS-I-6 (T^®^C) mutation ranks as the third most common mutation causing β-thalassemia in Southeast Brazil and it mainly affects individuals of Italian origin [15]. According to a study conducted in the southern part of the West Bank of the Palestinian Authority, the clinical manifestations of the homozygous IVS-I-6 individuals varied greatly, ranging from the usual transfusion-dependent thalassemia major phenotype to the non-transfusion-dependent thalassemia intermedia phenotype. The IVS-I-6 mutation may be very old, based on its frequency in certain West Bank groups [16].

**Fig. 1:**
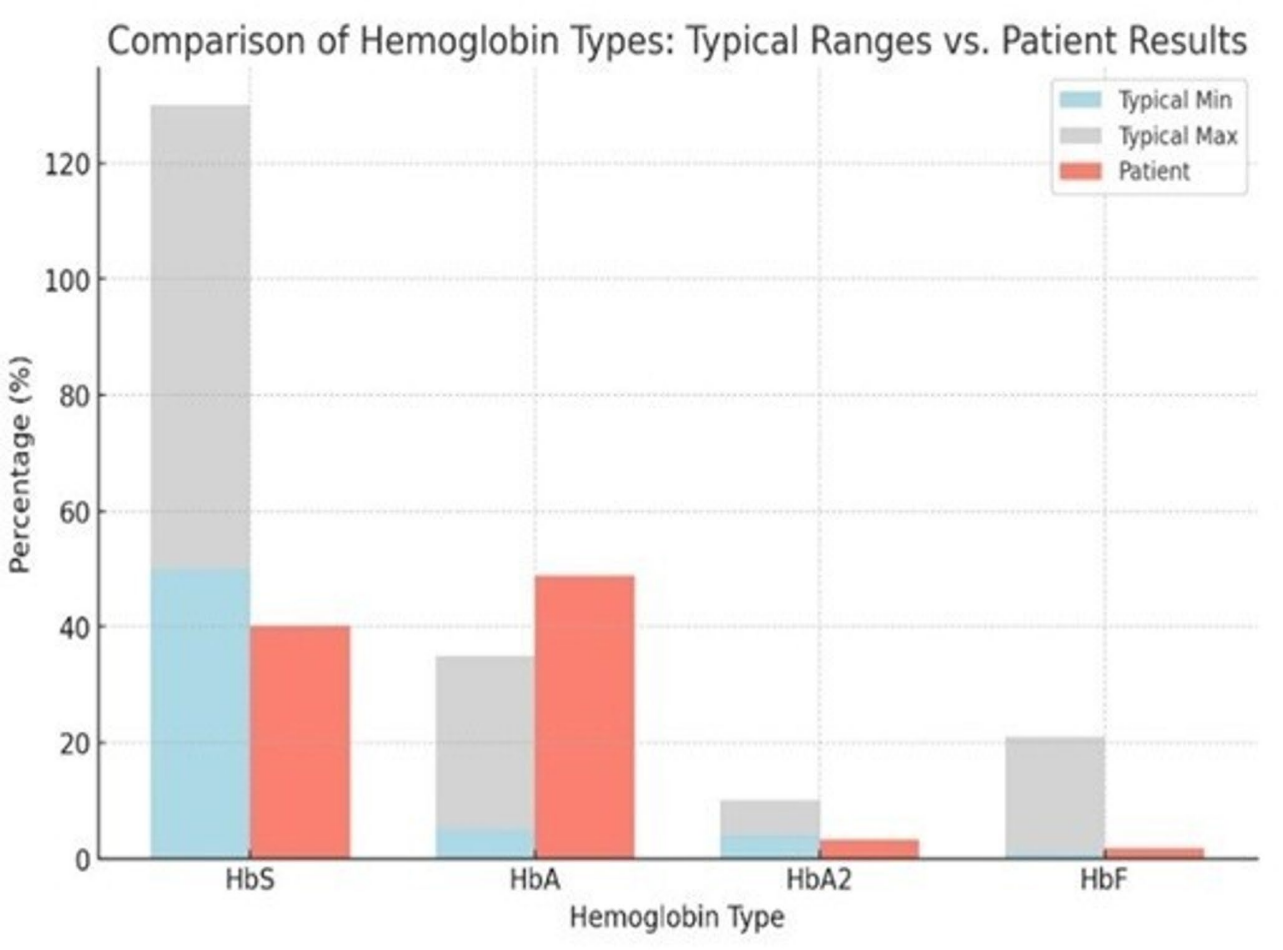
HbS: Although the patient's HbS level is slightly below the typical range (40.2% vs. 50–80%), it remains consistent with the diagnosis of HbSβ⁺ thalassemia. HbA: According to the data, the patient's level (48.8%) is rather high and significantly exceeds the average level (5-30%). HbA2: The patient's level (3.3%) is lower compared to other patients (it should be between 4-6%). HbF: The patient's level (1.8%) occurs exclusively in the lower end of the common 1-20% range

Figure 1 shows general values for HbSβ + thalassemia compared to our patient’s numbers. In detail, the chart shows:

The patient’s hemoglobin electrophoresis revealed a profile that differed from what is expected for HbSβ + thalassemia, according to Thein [17], lacks the actuality of high HbS and low HbA. This deviation indicates variability in the disease’s expression, implying that diagnosis and management of the condition should not be generalized but ought to be tailored to address individual genetic differences, as stated by Delicou et al. as well [8].

Sickle cell anemia typically manifests during the second half of the first year of life, whereas HbSβ-thalassemia usually becomes apparent at puberty [18]. Nevertheless, even patients with milder forms of SCD, such as HbSC and HbSβ+, can also develop vaso-occlusive crises and hemolytic anemia and all of the severe and life-threatening complications seen in sickle cell anemia (SCA) [18]. Bone marrow necrosis is known to affect patients with SCD, but there are also reports of its occurrence in those with HbSβ-thal; consequently, it is rather unusual to observe bone marrow infarction in a patient with HbSβ-thal [19]. Other rare presentations like multiorgan dysfunction, orbital compression syndrome, and fatal splenic sequestration crisis have also been reported in these patients [19].

Most patients with HbSβ + thalassemia are identified early in life due to anemia of moderate to severe intensity, pain crises, and systemic complications.In this case, the patient remained asymptomatic until the age of 40, which is highly unusual and could suggest the possible role of genetic modifiers or environmental factors in downregulating disease expression. The specific manifestations of our patient included back pain and anemia, which are common in sickle cell crises [20, 21]. Previous laboratory tests conducted in 2013, when the patient was 30 years old, showed normal parameters (Hgb: 13 g/dl, MCV: 94.2 fL, MCH: 31.6 pg). By 2015, when she was 32 years old, there was a mildly affected complete blood count showing microcytic hypochromic anemia (Hb: 11.6 g/dL, MCV: 70.6 fL, MCH: 22.2 pg) (see Table 2) but no further investigations were initiated as these blood tests were part of a routine check-up requested by the patient, who was not seeking medical care at the time.

Delayed diagnosis of hemoglobinopathies, particularly SCD, significantly impacts patients’ quality of life. Many patients report multiple consultations for characteristic symptoms yet remain undiagnosed [22]. Given the high prevalence of hemoglobinopathies in our country, the median age of death in those patients (the last published results of a cohort in Greece show a median age of 50.0 years old for thalassemia and 58.49 for SCD, *p* < 0.001), as well as the consequences of delayed diagnosis, evaluating the cost-effectiveness of universal screening may be warranted, particularly when prenatal screening has not been performed [8]. For example, in the United States, newborn screening for SCD, along with 29 core conditions, has been mandatory for decades [2]. Moreover, a delayed diagnosis under certain circumstances, such as pregnancy, can be troubling [23]. Socioeconomic factors have been implicated in delayed SCD diagnosis, even in lifelong symptomatic patients, such as a 52-year-old Nigerian woman [23–25]. In contrast, our patient remained asymptomatic for 40 years before diagnosis, making this case, to the best of our knowledge, unique.

The more severe forms of thalassemia, along with sickle cell anemia, require intensive medical treatment throughout the patient’s life. However, advancements in transfusion, iron chelation, and bone marrow transplantation therapies have significantly improved both life expectancy and quality of life in recent years [26]. Other medications, such as hydroxyurea, are also given. Hydroxyurea not only increases HbF in SCA but also exerts additional effects. It reduces inflammation by lowering neutrophil and platelet counts, increases the flexibility of red blood cells by elevating MCV, and increases blood flow to prevent vaso-occlusive crises. It may also enhance circulation by releasing nitric oxide, which makes it a first-line treatment for SCA [27]. In a study presented by Di Maggio et al., the percentages of patients using hydroxyurea who experienced increases in HbF levels and in MCV were similar among groups composed of those with HbSS, HbSβ0, and HbSβ+ [28]. Other disease-modifying treatments for SCD include voxelotor, l-glutamine, and crizanlinumab [29, 30]. Allogeneic hematopoietic stem cell transplantation is the only curative treatment now available and recommended by guidelines, enabling patients to become transfusion independent, but it is not without risks [31, 32]. Over the past three decades, research in gene therapy has evolved significantly, leading to FDA approval of two different gene therapies in 2023 for SCD patients > 12 years old with recurrent vaso-occlusive crises: exagamglogene autotemcel (Casgevy™) employs CRISPR/Cas9 gene editing technology to downregulate *BCL11A*, thus inhibiting transition from HbF to HbA. Lovotibeglogene autotemcel (Lyfgenia) is a lentiviral vector expressing a new HbAT87Q hemoglobin variant, comparable to normal HbA. Yet, the long-term impact of gene editing treatments remains unknown [33].

Different guidelines and practical recommendations on the management of thalassemias and SCD have been issued and include specific recommendations for managing individuals with HbSC and HbSβ + thalassemia (Table 3) [34]. Τhe International Collaboration for Transfusion Medicine Guidelines stresses that individuals with SCD or thalassemia should in most cases be transfused with ABO, Rh (D, C, E), and K matched RBCs to minimize the risk of alloimmunization. In patients who already have clinically relevant alloantibodies, a history of hemolytic transfusion reactions, or autoantibodies, it is strongly suggested that extended matching RBCs (with antigens such as Fya, Jka, and S) be employed. Although there is low general quality of evidence, recommendations have been increasingly reinforced over time, from “weak” to “strong” support for increased antigen matching to reduce risk for further alloimmunization and improve patient outcomes [35, 36].

Pain management, often overlooked by physicians unfamiliar with hemoglobinopathies, is crucial—particularly in acute vaso-occlusive crises—as it significantly contributes to morbidity and impacts patients’ quality of life [36]. Notably, pain was the primary reason for our patient’s initial consultation.

**Table 3:** Recommendations for managing individuals with HbSC and HbSβ + thalassemia [34]

Category	Recommendations
Infection Prevention	Individuals with HbSC and HbSβ + thalassemia have a lower risk of life-threatening infections due to normal or minimally impaired spleen function in infancy. However, older children and adults with any SCD genotype face increased bacterial infection risks. Penicillin prophylaxis can be discontinued in children with HbSC or HbSβ + thalassemia unless they have had a splenectomy (Weak Recommendation, Low-Quality Evidence).
Management of Splenic Sequestration	Splenic sequestration in individuals with HbSC and HbSβ + thalassemia often occurs later in childhood or adulthood and is typically associated with severe pain from splenic infarction, confirmable by imaging.
Transfusions	For individuals with HbSC or HbSβ + thalassemia and acute chest syndrome (ACS), transfusion decisions should be made in consultation with an SCD specialist (Strong Recommendation, Low-Quality Evidence).
Hydroxyurea Use	For individuals with HbSβ + thalassemia or HbSC who suffer from recurring sickle cell-associated pain that impacts daily activities or quality of life, consult a sickle cell specialist for potential hydroxyurea therapy (Moderate Recommendation, Low-Quality Evidence).
Procedures with General Anesthesia	Before any surgical procedure involving general anesthesia, adults and children with HbSC or HbSβ + thalassemia should consult a sickle cell expert to determine if a full or partial exchange transfusion is necessary (Moderate Recommendation, Low-Quality Evidence).
Diagnostic Examinations	Transcranial Doppler screening is not recommended for children with HbSβ + thalassemia or HbSC (Strong Recommendation, Low-Quality Evidence).

Guidelines for hydroxyurea use, RBC transfusion, and antibiotic prophylaxis are still limited, and there is no agreement on the management plan [37]. The use of hydroxyurea in combination with tailored transfusion protocols was essential in managing our patient’s symptoms and preventing further complications.

During the following six months, the patient was closely followed up, showing clinical improvement—with resolution of palpitations, dizziness, and back pain—and significant improvement in her general exercise tolerance and well-being. Her laboratory values returned to normal, with hemoglobin increasing to 10.2 g/dL. Follow-up imaging revealed neither signs of bone marrow infarction nor other complications. She continued hydroxyurea treatment and was also referred for genetic counseling for future management and planning of family needs.

In conclusion, the presented case involves compound heterozygosity for SCD and β-thalassemia, emphasizing the importance of recognizing atypical presentations of SCD, including its potential onset in adulthood, even as late as the age of 40. A delayed diagnosis is often more challenging and requires high clinical suspicion, underscoring the potential need for screening protocols in populations with a high prevalence of these conditions. Further research on the management of double heterozygous sickle-beta-thalassemia and its phenotypic heterogeneity is essential to improve treatment protocols and outcomes for patients with similar complex conditions.

## Data Availability

No datasets were generated or analysed during the current study.
